# Descriptive Analysis of Patients’ EMS Use Related to Severity in Tokyo: A Population-Based Observational Study

**DOI:** 10.1371/journal.pone.0059738

**Published:** 2013-03-20

**Authors:** Toshikazu Abe, Shinichi Ishimatsu, Yasuharu Tokuda

**Affiliations:** 1 Department of Emergency Medicine, Mito Kyodo General Hospital, University of Tsukuba, Mito City, Ibaraki, Japan; 2 Department of Emergency and Critical Care Medicine, St Luke’s International Hospital, Tokyo; 3 Institute of Clinical Medicine, Graduate School of Comprehensive Human Sciences, University of Tsukuba, Mito City, Ibaraki, Japan; University of Pittsburgh Medical Center, United States of America

## Abstract

**Introduction:**

Few studies are available on the clinical characteristics of patients using emergency medical transports in Japan. In this study, we aimed to investigate reasons for emergency medical transports and their relation to clinical severity.

**Methods:**

We conducted a 3-year population-based observational study of patients transported by ambulance to emergency departments (ED) in the capital of Japan, Tokyo, which has a population of about 13 million. Demographic data, reasons for transport, and the severity of initial assessment at ED were recorded. Logistic regression was used to determine the odds of the clinical severity of each reason for transport.

**Results:**

The number of emergency medical transports in the three-year study period was 1,832,637. Mean age was 53±26. Males were 976,142 (53%). Overall, 92% of all transported patients were in a mild or moderate medical state and patients with the 17 most frequent reasons for transport occupied 82% (1,506,017) of all transports. Pain was the most frequent reason for transport, followed by traffic accident. Considering all the patients and their reasons for transport, patients whose reason was pain or a traffic accident (29% of all patients) were in a relatively mild state compared with patients with other reasons for transport. Patients in an altered mental state in the prehospital setting (6.8% of all patients) were in a more severe medical state than other patients.

**Conclusions:**

In Tokyo, Japan, 92% of transported patients were in a mild or moderate medical state. In particular, most patients from traffic accidents were in a mild state, even though traffic accidents were the second most frequent reason for transport. Patients in an altered mental state were most likely to be in a severe medical state.

## Introduction

One of the current crucial problems of emergency medicine in Japan is the increased number of medical transports. The number of emergency medical transports in 2008 was one and a half times the number in 1998. Therefore, the mean time of ambulance arrival to scenes was 7.7 min in 2008, 1.7 min longer than the mean time in 1998. [1] Tanigawa et al. reported that the majority of emergency calls were for minor injuries or illness, which compromised arrival times to sites of severe illness or injury. [2] There are multiple episodes of patients with mild illnesses using ambulances like taxis because they are free of charge. It is also related to the increased number of medical transports due to the rapidly aging population of Japan. [3].

Finding alternate means for these patients to seek care is currently one of the biggest topics of emergency medicine in Japan. However, there have been few studies that have examined the clinical characteristics of patients using emergency medical transport in Japan. To enhance more appropriate use of emergency medical transport, it is important to understand the nature of reasons for transports in Japan. Most studies in other countries investigated the different uses of emergency medical transports by people with different types of insurance because economics is more related to this problem. [4] In contrast, Japan has a universal national health insurance system [5] that has many reasonable benefits for patients including universally available free ambulances. Of course, a sole source pay system may lead towards adverse outcomes for society. Therefore, even though insurance system issues are controversial, we could investigate the nature of reasons for transports without issues related to insurance coverage.

In this study, we aimed to investigate the differences in clinical severity based on reasons for transport. With these results, future interventions could be implemented to enhance more appropriate use of emergency medical transport in Japan.

## Methods

### Ethics Statement

The ethics committee at our institution does not require its approval for observational studies using anonymous data previously collected for routine operations. Also, informed consent from each patient is waived for using anonymous data according to the informed consent guidelines in Japan. [6] We obtained these anonymous data with the permission of the Tokyo Fire Department. [1] We conducted this research according to the principles expressed in the Declaration of Helsinki.

### Study Population

This was a population-based prospective observational study conducted in Tokyo, the capital of Japan, with a population of approximately 13 million in 2011, a land area of 2187 square kilometers, and a temperate climate. The Tokyo Metropolitan Fire Department oversees a single-tiered system covering the entire metropolitan area, with basic life-support ambulances based throughout at 80 fire stations. Ambulance service is free of charge and staffed by non-physician emergency medical technicians (EMSs). [7] Using information provided by all the emergency departments (EDs) in Tokyo, the ambulance staff keep a digitized record of initial medical data for all patients transported to an ED. The Tokyo Fire Department’s digitized registry contains all EMS transports in Tokyo.

### Data Collection

Data were reviewed for all patients transported to hospital EDs in Tokyo during the 3-year period from 1 January 2006 through 31 December 2008, using the Tokyo Fire Department’s digitized registry of data on transported patients. We included only patients who were transported to hospital EDs by ambulance, including those involved in traffic accidents and fires. Our data set did not include helicopter transports or walk-ins or other means of reaching an ED. Multiple transports of the same patient were included. For each patient, we collected demographic data (age and sex), reason for transport, and initial impression of medical severity according to the emergency physicians. In the reasons for transport, we divided all patients into two groups: internal causes and external causes. Pain, altered mental state, dyspnea, weakness, fever, dizziness, vomiting, seizure, nausea, walking difficulty, palpitation, and numbness were reasons with internal causes. We also selected traffic accidents as a subgroup for analysis in external causes. Severity was classified as mild (outpatients), moderate (admitted but not severe), severe, critical, or death. Death data included those before and during transportation to the hospital. Categorization of severity of illness was determined by emergency physicians at ED. It was a clinical decision based on their initial assessment including vital signs. However, we could use only their categorization of severity in the dataset. Details of severity such as vital signs were not included in the dataset because they omitted them when they recorded in the database. We also collected the time data on when patients called the ambulance. Hospital admission data were collected from the EDs. The confidentiality of patient data was carefully protected. These data were originally recorded in paper-based registration by emergency physicians and then EMSs collected and recorded them electronically later.

### Statistical Analyses

For characteristics of patients, categorical variables are shown by frequency and percentage. Continuous variables are shown as mean±SD. Age was divided into 4 groups: infant-toddler (0–5 y.o.), child (6−17 y.o.), adult (18–59 y.o.), old (60–84 y.o.), and oldest old (85–99 y.o.). We demarcated four seasons in Japan: Spring (March - May), Summer (June - August), Fall (September - November), and Winter (December - February). Time of ambulance call was divided into 4 periods: early morning (0–6), morning (6–12), afternoon (12–18), and night (18–24). We analyzed the number of patients by time period and season of the ambulance call. Based on the ambulance transport data, the 17 most frequent symptoms/events comprising more than 82% of all transports were determined. Because each of the other symptoms/events was identified only in a few patients (less than 0.9% of total), we omitted these other reasons for transport from the list. We divided the severity into binary outcome (moderate and severe). Because patients in mild (outpatients) or moderate (admitted but not severe) states were considered not in need of urgent care, they could choose other methods of transports such as a private ambulance which cannot use the authorized urgent signs. We used these variables (moderate vs. severe) as the dependent variable. Univariate analysis was performed using logistic regression to examine the significance of each predictor. To compare binary variables between the groups, we show effect size using confidence intervals (CIs) because of the large database. For the final modeling, independent variables were age, group, gender, time period of call, and frequent reasons for transport. We compared the model of each variable selection (e.g. pain) with the model of all variables because characteristics of all variables would generalize those of patients in ambulance transports. The dependent variable was binary severity of initial evaluation. Using those independent variables, a multivariable logistic regression model was constructed for independent predictors of patients with mild states; odds ratios of 95% CI and p-values were estimated. All P values were 2-sided and P<0.01 was considered to be statistically significant because of the large database. Statistical analyses were performed using STATA 11.2 (Stata Corp, TX, USA).

## Results

The numbers of emergency medical transports were 626,543 in 2006, 623,012 in 2007, and 583,082 in 2008. The patients used ambulances most frequently in December [172,580 (9.4%)], and least frequently in June [142,991 (7.8%)]. The patients used ambulances most in the afternoon [555,900 (30%)] and least in early morning [274,265 (15%)]. (Table 1) Mean age was 53±29. In the age category, 91,523 (5.0%) of patients were from infant to toddler, 73,631 (4.0%) were children, 823,172 (45%) were adults, 660,302 (36%) were old, and 184,009 (10%) were oldest old. Males were 976,142 (53%). (Table 1).

**Table 1:** Characteristics of emergency medical transports in Tokyo.

Age		Number	Percentage
Infant-toddler	1–5	91,523	5.0%
Child	6–17	73,631	4.0%
Adult	18–59	823,172	45%
Old	60–84	660,302	36%
Oldest old	85–99	184,009	10%
Total		1,832,637	100%
**Season**	**Month**	**Number**	**Percentage**
Spring	March	157,959	8.6%
	April	148,327	8.1%
	May	149,429	8.2%
Summer	June	142,991	7.8%
	July	154,458	8.4%
	August	155,192	8.5%
Fall	September	143,464	7.8%
	October	150,829	8.2%
	November	150,470	8.2%
Winter	December	172,580	9.4%
	January	161,678	8.8%
	February	145,260	7.9%
Total		1,832,637	100%
**Time zone**	**Time**	**Number**	**Percentage**
Early morning	0–6	274,265	15%
Morning	6–12	492,761	27%
Afternoon	12–18	555,900	30%
Night	18–24	509,711	28%
Total		1,832,637	100%
There were no missing data.			

Table 2 shows the transport rates for the 17 most frequent reasons for emergency medical transport in Tokyo. Patients with the 17 most frequent reasons for transport comprised 82% (1,506,017/1,832,637) of all transports. Pain was the most frequent reason for transport, followed by traffic accident. Cut wound was the least frequent reason for transport, followed by blow wound. The each of other symptoms than 17 most frequent reasons comprised less than 1.0% of total transports.

**Table 2:** Transport rate for 17 most frequent reasons for ambulance transport in Tokyo.

Symptom	Number	Percentage of Total Transportation	95CI	
			Lower	Higher
Pain	330,011	18%	18%	18%
Traffic Accident	201,693	11%	11%	11%
Fall	183,196	10%	10%	10%
Altered Mental State	124,341	6.8%	6.7%	6.8%
Dyspnea	99,801	5.4%	5.4%	5.5%
Weakness	92,479	5.0%	5.0%	5.1%
Fever	83,450	4.6%	4.5%	4.6%
Dizziness	78,091	4.3%	4.2%	4.3%
Vomiting	72,505	4.0%	3.9%	4.0%
Seizure	58,923	3.2%	3.2%	3.2%
Nausea	43,866	2.4%	2.4%	2.4%
Walking Difficulty	32,875	1.8%	1.8%	1.8%
Palpitation	23,384	1.3%	1.3%	1.3%
Downfall	22,728	1.2%	1.2%	1.3%
Numbness	21,761	1.2%	1.2%	1.2%
Blow	19,752	1.1%	1.1%	1.1%
Cut	17,161	0.9%	0.9%	1.0%
Sum	1,506,017	82%		
There were no missing data.				

Regarding the severity of all transported patients, 1,090,499 (60%) patients were in a mild state (discharged from EDs without hospital admission), and 593,348 (32%) were in a moderate state (admitted but not severe). Thus, 92% of all transported patients were in a mild or moderate state. The state was severe in 92,310 (5.0%), critical in 43314 (4.2%), and dead in 13,166 (0.7%). (Table 3).

**Table 3:** Severity of patients in ambulance transports in Tokyo.

Severity		Number	Percentage
Moderate	Mild	1,090,499	60%
	Moderate	593,348	32%
Severe	Severe	92,310	5.0%
	Critical	43,314	2.3%
	Death	13,166	0.7%
Total		1,832,637	100%
There is no missing data.			

Table 4 shows the results of univariate analysis using a logistic regression model including characteristics of the ambulance transports. Patients in an altered mental state or with dyspnea were the most likely to be admitted to hospital. Patients with dizziness were in a relatively mild state compared with those with no dizziness. In age category, oldest old and old were the most likely to be admitted to hospitals. In time zone of morning patients were the most likely to be admitted to hospitals.

**Table 4:** Univariate analysis using logistic regression for severe patients by reason for ambulance transport.

Characteristics		beta	beta of SE	OR	95CI of OR	
					Lower	Higher
Age		0.03	0.000	1.03	1.03	1.03
Gender (Male vs Female)		−0.29	0.006	0.75	0.74	0.75
Pain		−0.55	0.010	0.57	0.56	0.59
Altered Mental State		1.81	0.008	6.08	5.99	6.18
Dyspnea		0.76	0.010	2.14	2.10	2.19
Weakness		−0.27	0.014	0.77	0.75	0.79
Fever		−0.66	0.018	0.52	0.50	0.53
Dizziness		−2.21	0.037	0.11	0.10	0.12
Vomiting		−1.03	0.025	0.36	0.34	0.38
Seizure		0.00	0.021	1.00	0.96	1.04
Nausea		−0.92	0.028	0.40	0.38	0.42
Walking Difficulty		−0.25	0.022	0.78	0.75	0.82
Palpitation		−1.11	0.041	0.33	0.30	0.36
Numbness		−0.68	0.037	0.51	0.47	0.54
		**Moderate (Number)**	**Severe (Number)**	**OR**	**95CI of OR**	
					**Lower**	**Higher**
Age Category	Infant-Toddler	89,633	1,890	Reference		
	Child	72,434	1,197	0.78	0.73	0.84
	Adult	785,491	37,681	2.28	2.17	2.38
	Old	581,716	78,586	6.41	6.12	6.71
	Oldest Old	154,573	29,436	9.03	8.61	9.47
Time Zone	Early Morning	253,985	20,280	Reference		
	Morning	448,161	44,600	1.25	1.22	1.27
	Afternoon	509,335	46,565	1.14	1.13	1.16
	Night	472,366	37,345	0.99	0.97	1.01
There is no missing data.						

We used a multiple logistic regression model adjusted for age, gender, and time period in the final model. Among those with the 17 most frequent reasons for transport, patients whose reason was pain or traffic accident (29% of all patients) were in a relatively mild state compared with those with the other reasons for transport. Patients in an altered mental state (6.8% of all patients) were in a more severe state than those with other reasons for transport. (Table 5) Regarding traffic accidents as a subgroup, pedestrians were in a relatively more severe state, compared to those who had been inside a car. (Table 6) A subgroup analysis of internal causes showed that those in an altered mental state or with dyspnea were more likely to be in a severe state. Patients with dizziness were in a relatively mild state compared with those with other internal causes. (Table 7).

**Table 5:** Multiple logistic-regression model for severity of severe, critical and death of ambulance transport patient.

Characteristics		beta	beta of SE	OR	95CI of OR	
					Lower	Higher
Age		0.03	0.00	1.03	1.03	1.03
Gender (Male vs Female)		−0.28	0.01	0.75	0.75	0.76
Time Zone	Early Morning	Reference				
	Morning	−0.09	0.01	0.91	0.90	0.93
	Afternoon	−0.18	0.01	0.84	0.82	0.85
	Night	−0.17	0.01	0.85	0.83	0.86
Pain		−1.35	0.01	0.26	0.25	0.26
Traffic Accident		−1.82	0.02	0.16	0.16	0.17
Fall		−3.33	0.03	0.04	0.03	0.04
Altered Mental State		1.02	0.01	2.78	2.74	2.82
Dyspnea		−0.04	0.01	0.97	0.95	0.98
Weakness		−1.05	0.01	0.35	0.34	0.36
Fever		−1.44	0.02	0.24	0.23	0.24
Dizziness		−3.00	0.04	0.05	0.05	0.05
Vomiting		−1.82	0.03	0.16	0.15	0.17
Seizure		−0.77	0.02	0.46	0.44	0.48
Nausea		−1.71	0.03	0.18	0.17	0.19
Walking Difficulty		−1.03	0.02	0.36	0.34	0.37
Palpitation		−1.91	0.04	0.15	0.14	0.16
Downfall		−1.50	0.04	0.22	0.21	0.24
Numbness		−1.47	0.04	0.23	0.21	0.25
Blow		−2.83	0.09	0.06	0.05	0.07
Cut		−0.76	0.04	0.47	0.43	0.50

**Table 6:** Subgroup analysis of traffic accident using logistic regression for severity.

Characteristics		beta	beta of SE	OR	95CI of OR	
					Lower	Higher
Age		0.03	0.000	1.03	1.03	1.03
Gender (Male vs Female)		−0.37	0.006	0.69	0.68	0.70
Traffic Accident	Pedestrian	−0.39	0.031	0.68	0.64	0.72
	Bicycle	−1.59	0.033	0.20	0.19	0.22
	Motorcycle	−0.69	0.030	0.50	0.47	0.53
	Car	−2.08	0.046	0.12	0.11	0.14

**Table 7:** Subgroup analysis of internal causes using logistic regression for severity.

Characteristics	beta	beta of SE	OR	95CI of OR	
				Lower	Higher
Age	0.03	0.000	1.03	1.03	1.03
Gender (Male vs Female)	−0.29	0.006	0.75	0.74	0.75
Pain	−0.55	0.010	0.57	0.56	0.59
Altered Mental State	1.81	0.008	6.08	5.99	6.18
Dyspnea	0.76	0.010	2.14	2.10	2.19
Weakness	−0.27	0.014	0.77	0.75	0.79
Fever	−0.66	0.018	0.52	0.50	0.53
Dizziness	−2.21	0.037	0.11	0.10	0.12
Vomiting	−1.03	0.025	0.36	0.34	0.38
Seizure	0.00	0.021	1.00	0.96	1.04
Nausea	−0.92	0.028	0.40	0.38	0.42
Walking Difficulty	−0.25	0.022	0.78	0.75	0.82
Palpitation	−1.11	0.041	0.33	0.30	0.36
Numbness	−0.68	0.037	0.51	0.47	0.54

## Discussion

Our results based on a study in Tokyo, Japan, indicated that 92% of all transported patients were in a mild or moderate state. Patients in an altered mental state were more likely to be in a severe medical state. In contrast, trauma patients, especially from a traffic accident, or fall, were likely to be in a mild or moderate state, and these patients comprised about 21% of all emergency medical transports. In particular, most patients from traffic accidents were in a mild state, even though traffic accident was the second most frequent reason for use of emergency medical transport.

In our study, most transported patients were brought to hospitals in spite of being in a less than severe state. 1,090,499 (60%) patients were discharged without hospital admission and 593,348 (32%) patients were admitted but their state was not severe. These numbers seem much higher compared to patients in the US [8], where many patients may get to the hospital by themselves or with the assistance of their family or an alternative way. These results imply that citizens in Tokyo might inappropriately overuse ambulances.

Although education of citizens is needed for more appropriate use of ambulances, there are some reasons for this result. They might have few available ways to get to a hospital because there were many older patients, since 46% of all the population was over 60 years old. Richardson et al. reported that having no other means of transportation was the main reason for use of emergency medical transports, especially in big cities. [9] Moreover, because the Japanese family has shifted to the nuclear family, people might ask other non-family people whether they should call an ambulance or not. Those who give advice are usually laypersons and may overestimate the seriousness of a patient’s illness and thus may recommend them to call an ambulance.

In Japan, transported patients with trauma were likely to be in a mild or moderate state. They comprised about one fourth of all ambulance transports. Especially, there were many patients from traffic accidents who were in a mild state. Ambulances are called to almost all traffic accidents in Japan. Even if casualties are obviously mild, a policeman usually calls an ambulance after police inspection of an accident site because it is an easy way to care for victims. Thus, many definitely mild casualties from traffic accidents are involuntarily brought to hospitals by ambulance. It might be more efficient to pre-select these patients to reduce ambulance transports, because triage could be performed for patients with trauma more easily than those with internal diseases. [10] In the US, many casualties from traffic accidents do not use an ambulance and instead go to a hospital by themselves. We may be able to use the information gained from this study and comparisons with other systems to make some policy changes before our ambulance system collapses.

Patients in an altered mental state in the prehospital setting were in the most severe states of all transports. Clinically it makes sense, because there are many severe illnesses in differential diagnoses which cause an altered mental state, including cerebrovascular diseases, sepsis and others. EMTs and emergency physicians need to give careful attention to patients in an altered mental state in the prehospital setting.

Triage is one effective way to reduce emergency medical transports. In Japan we also have a field triage system like that in the US, [11] however, it is only a decision-making tool which hospital EMS can choose. Our system never allows EMS to reject a patient, even if the patient is in a very mild state. Thus, our system cannot reduce the number of transports by field triage. Phone triage has been experimented with in Yokohama since 2008. Oshige et al. reported that a patient’s life threat risk was quantitatively assessed at the moment of the emergency call with a moderate level of accuracy. However, phone triage in Yokohama is just a pilot program and it has not been implemented in Japan yet. [12] We may need to consider multiple levels for more effective triage such as the system of Alameda County, CA. [13] It is very difficult to determine appropriateness for ambulance transport and thus prehospital triage should be carefully performed if it is planned. Appropriate utilization should be equal to reduce inappropriate utilization but it may put some patients vulnerable. [14], [15] For example, some patients with severe back pain should call an ambulance even if a urinary stone is the most likely cause, because they might have aortic dissection.

Our study has some strengths. First, our data were not influenced by economic issues including insurance and costs, because in Japan we have a universal national insurance system and patients receiving welfare benefits do not need to pay for health care. Citizens do not need to pay any charge for ambulance transport and they do not have to pay any extra cost over about ¥ 80,000 (1USD = ¥ 80) per a month even for expensive hospital care. Second, there were no missing data in our study. It might be rare in for such a large size database.

There are limitations in our study. First, estimation of patients’ severity was based on the initial impression of emergency physicians. Thus, it would have introduced a bias because it was just subjectively determined. Follow-up data on these patients were not available. The severity of a patient’s state could change later. Also, acuity level, like hospital admission and discharge, is not only decided by severity but also by hospital policy. Second, the data in this study were solely based on the total number of patients. Some patients might have been brought to the hospital twice. Bounce-back patients are often in a severe state. Third, we investigated only reasons for transport and age, gender, time, seasons, and states of severity. There must be other potential variables to calling an ambulance. For example, there could be more important determinants for acuity like vital signs even though they were not available in this database. Fourth, these results might be more apparent in a big city such as Tokyo. Generalization to other settings like- countryside, semiurban areas etc. and other countries should be made with caution. Fifth, the number of patients who did not use EMS would be needed in order to accurately evaluate whether EMS was appropriately utilized among less ill patients. But it is likely there is a large number of mildly ill patients who call an ambulance because ambulances are free of charge and because universal national health insurance is maintained in Japan. We believe that our results showed our objective appropriately.

In conclusions, most transported patients were in a mild or moderate medical state in Tokyo, Japan. Trauma patients including from a traffic accident or a fall were less severe and could have gone to the hospital by themselves. Especially, there were many patients from traffic accidents who were in a mild medical state and thus they could be a candidate group for being effectively triaged in the prehospital setting. In contrast, patients in an altered mental state were more likely to be in a severe medical state. Further studies are needed to develop novel interventions to reduce emergency medical transports in Japan.
